# Formulation, Characterization and Optimization of Liposomes Containing Eicosapentaenoic and Docosahexaenoic Acids; A Methodology Approach

**Published:** 2014

**Authors:** Zahra Hadian, Mohammad Ali Sahari, Hamid Reza Moghimi, Mohsen Barzegar

**Affiliations:** a *Faculty of Agriculture, Tarbiat Modares University and Academic Staff of National Nutrition and Food Technology Research Institute, Shahid Beheshti University of Medical Sciences,*; b *Department of Food Science and Technology, Faculty of Agriculture, Tarbiat Modares University, Tehran, Iran.*; c *Department of Pharmaceutics, Faculty of Pharmacy, Shahid Beheshti University of Medical Sciences, Tehran, Iran. *; d *Department of Food Science and Technology, Faculty of Agriculture, Tarbiat Modares University, Tehran, Iran.*

**Keywords:** Preparation, Characterization, Liposome, DHA, EPA

## Abstract

Omega-3 fatty acids (FAs) have been shown to prevent cardiovascular disease. The most commonly used omega-3 fatty acids like eicosapentaenoic acid (EPA) and docosahexaenoic acid (DHA) are highly vulnerable to oxidation and therefore, have short shelf life. Recent advances in nanoliposomes provided a biocompatible system for stabilizing omega-3 FAs. Several methods could be implemented to prepare nanoliposomes. To the best of our knowledge, the performances of these methods in preparation omega-3 FAs have not been examined. Nanoliposomes were prepared by thin film hydration followed by one of the following methods: 1- extrusion, ultrasonic irradiation; 2- bath sonication; 3- probe sonication; or 4- combined probe and bath sonication. The size of liposomes obtained from methods 1 to 4 were 99.7 ± 3.5, 381.2 ± 7.8, 90.1 ± 2.3, and 87.1 ± 4.10 nm with zeta potential being -42.4 ± 1.7, -36.3 ± 1.6, -43.8 ± 2.4, and 31.6 ± 1.9 mV, respectively. The encapsulation efficiency (EE) for DHA was 13.2 ± 1.1%, 26.7 ± 1.9%, 56.9 ± 5.2% and 51.8 ± 3.8% for methods 1 to 4, respectively. The corresponding levels for EPA were 6.5 ± 1.3%, 18.1 ± 2.3%, 38.6 ± 1.8%, and 38 ± 3.7%, respectively. The EE for DHA and EPA of liposomes for both methods 3 and 4 increased significantly (p<0.05). Propanal, as the major volatile product formed during liposomal preparations, amounts from 81.2 ± 4.1 to 118.8 ± 2.3 μg/Kg. The differential scanning calorimetry (DSC) study showed that DHA and EPA influence the phase transition temperature of small unilamellar vesicles (SUVs) of dipalmitoyl phosphatidyl choline (DPPC). Transmission electron microscopy (TEM) images of liposomes stained with uranyl acetate showed that the liposomes were spherical in shape and maintain high structural integrity. In conclusion, probe ultrasound of pre-formed liposomes facilitates significant loading of DHA and EPA into the nanoliposomal membrane.

## Introduction

Our understanding of the role of omega-3 FAs in secondary prevention of coronary heart disease (CHD) is becoming clearer (1, 2). Among different ethnicities, it has been shown that intake of about 1 g per day clinically confer meaningful benefits to patients with preexisting CHD (3, 4). Thus, it seems appropriate to consider recommending increased omega-3 FAs intake both for patients with CHD and for patients being high risk for CHD. In fact, after 25 years of omega-3 FAs research, there is sufficient evidence to support a recommendation that patients with or at risk for CHD increase their EPA and DHA intake (5). Since the American Heart Association (AHA) first released its scientific advisory, “fish consumption, fish oil, lipids and coronary heart disease,” a plethora of evidence relating to the beneficial effects of omega-3 FAs, in areas other than heart disease, has emerged (6). It also has been demonstrated that two omega-3 fatty acids including DHA and EPA are essential for the physiologic function of neuronal cell membrane. Brain lipids are highly enriched in PUFA (7, 8). Benefits have been documented in both dietary and supplementation settings. However, the scientific statements of the AHA as well as other studies indicate that fish and seafood are potentially a major source of human exposure to various environmental contaminants (9). Fish contaminated with dioxins, polychlorinated biphenyls (PCB), and methyl-mercury can harbor risks to health (10, 11). The high degree of unsaturation renders the omega-3 fatty acids highly susceptible to oxidation and it results in loss of nutritional value and development of flavors that are unacceptable to consumers. Therefore, lipid oxidation is the most critical parameter affecting the shelf life. There is considerable interest towards development of PUFA-delivery systems that would result in sufficient oxidative stability. Different strategies such as encapsulation and antioxidant are used to prevent DHA and EPA from oxidation, decreases the odors of volatile oxidation products, and make them palatable (12, 13). Recent studies have shown that production of nanoliposomes could be considered as an effective technology for encapsulation of bioactive compounds, as well as for enhancing their stability and bioavailability (14, 15). Liposomes are microscopic phospholipid bubbles with a bilayer membrane structure. They have unique structure and physical and chemical properties enabling them to incorporate a wide spectrum of functional components in their interior size (16, 17). There are ongoing needs for techniques to be implemented in the efficient and rapid enrichment of drugs in liposomes (18, 19). The primary goal of this study was to encapsulate DHA and EPA in nanoliposomes by implementing four different methods: extrusion, probe sonication, bath sonication, and combined probe and bath sonication. We then, compared different methods in respect of size, ξ potential, encapsulation efficiency and volatile compounds. Moreover, the morphology of liposomes and physical properties of the DPPC bilayer were studied by treating DHA and EPA. 

## Experimental

*Materials*

We purchased 1, 2-dipalmitoyl-sn-glycero-3-phosphocholine (DPPC, purity 95%) from Lipoid (Lipoid KG, Germany). The lipid composition of DPPC has been identified using the gas chromatography (GC). DHA (purity 99.0%), EPA (purity 99.0%), phosphate buffer solution, sephadex G-50 and 2, 5-dimethylfuran was prepared from Sigma Chemical Co. (St. Louis, MO, USA). The purity of DHA and EPA was verified by nuclear magnetic resonance (NMR). Polycarbonate membranes were obtained from Whatman (Maidstone, UK). Propanal, pentanal, hexanal, heptanal, n-dodecane (purity 99%), boron trifluoride 12% (BF_3_), pentadecanoic acid, and all other chemicals used were of analytical grade and purchased from Merck (Chemical Co. Darmstadt, Germany). 

*Preparation of liposomes*

Multilamellar vesicles (MLV) were prepared using the conventional film method (18). A known amount of DPPC (25.7 mg mL^-1^) was dissolved in chloroform and methanol (at the ratio of 2: 1) and deposited as a thin film in a round-bottom flask by rotary evaporation under reduced pressure at 50 ºC above the DPPC transition temperature (Tc) is about 42 ºC. The residual traces of solvents were removed by a further evaporation under N_2_ stream. The resulting dried lipid film was hydrated for about 2 h by addition of phosphate buffer (0.05 M, pH 7.4) and glass beads (5 g) with mild agitation at 50 ºC. These MLVs were extruded through polycarbonate membranes with defined pore size or sonicated to get uncharged liposomes of the preferred size.

*Extrusion*

The MLVs were three times prefiltrated through a membrane with 0.2 µm pores size using discontinuous extruder (Liposo-Fast™ Avestin Inc., Ottawa, Canada). To allow the formation of smaller vesicles (~100-110 nm), this was followed by extrusions through double stacked membranes with 100-nm pores. The procedure was repeated three times at low extrusion pressure (200 psi). During all the extrusions, the temperature was maintained at least 10 ºC above the glass transition temperature of the DPPC (20).

*Sonication*

The MLV dispersion is placed in a glass vial and sonicated by bath, probe, or combined bath and probe methods. In method 2, MLV suspension was sonicated for 20 min in a bath-type sonicator in the continuous mode at 37 ºC (Tecno-Gaz; Tecna3 S.P.A, Bologna, Italy). In method 3, MLV dispersion was sonicated using a 20-kHz low-frequency ultrasonic processor (Hielscher UP200H, Germany). The ultrasonic titanium probe (7-mm diameter) was immersed in a glass vial, containing 5 mL of liposomes dispersion, at 20 mm of depth (diameter 18 mm and height 70 mm). A pulsed duty cycle of 6s on, 4s off was used with power delivery 30% for 10 min by section of 5 min. The sample vial was kept in a temperature-controlled water bath and its temperature was monitored throughout the experiment to prevent heat damage (4 ºC). Both probe and bath sonication procedures were carried out in the same way as the previous methods. The suspension prepared was sonicated in bath sonicator for 10 min and subsequent by probe for 30 min (21, 22).

*EPA and DHA loading*

EPA and DHA were dissolved in ethanol to the final concentrations of 10 and 5 mmol L^-1^, respectively. For loading two separate solutions of EPA and DHA, after drying under a stream of N_2,_ omega-3 FAs were added to the suspension of liposomes. The total concentration of phospholipid and free FAs were kept constant; 35 and 15 mmol L^-1^, respectively. In the extrusion method, EPA and DHA loaded in liposomes after hydration of dry thin lipid layer. After downsizing of MLVs by bath, probe, or combined bath and probe methods, omega-3 FAs loaded into pre-formed suspension of the liposomes, and then incubated at the ambient temperature for 2 hours. All prepared liposomes were refrigerated at 4 ºC for further analysis (23).

*Particle size and *zeta *potential measurement*

The hydrodynamic diameter, size distribution (polydispersity index; PDI), and Zeta potential ofall liposome preparations were measured by the dynamic light scattering (Nano ZS Malvern Zeta Sizer model 1000HSa, UK) technique at 25 ºC, using a He-Ne laser of 633 nm and a detector angle of 173 ˚C. Three independent measurements were performed for each sample. The Malvern measures the time-dependent fluctuations of light scattered by the liposomes and uses it to calculate the average size and polydispersity of the liposomes. Samples were analyzed 24 h after preparation. Liposomes were appropriately diluted with the aqueous phase of the formulations prior to the measurements. The particle size values given are averages of three measurements and are expressed as mean Zeta potential (24).

*Determination of DHA and EPA encapsulation efficiency*

EPA and DHA encapsulated in the bilayer DPPC were separated from free EPA and DHA on sephdex-G50 column. The filtration columns were prepared and packed. The gel column was prepared first by boiling about 1 g of sephdex-G50 fine powder in 20 mL of DI-water for an hour in a covered beaker. The gel was cooled to the room temperature and packed into 80% of the total column volume. Encapsulation efficiency of EPA and DHA were calculated indirectly using size exclusion chromatography method as described in the literature (25). Briefly, liposome suspensions/free EPA and DHA (1 mL) were eluted by phosphate buffer (pH 7.4) in a sephadex-G50 column (1.5 × 20 cm), and encapsulated EPA and DHA were separated from free fatty acids and then the vesicles of the eluate were collected from the first 15 mL. The concentration of entrapped EPA and DHA were determined by gas chromatography (GC). Encapsulation efficiency (EE) was calculated using the following formula (26):

$\mathrm{EE\%}=\frac{C}{C_{{}^{\circ}}}\times 100$

Where C is the concentrations of entrapped EPA and DHA in the elute, and C_0_ is the initial amount of EPA and DHA used for preparing the liposomes.

*GC analysis of fatty acids*

The total lipid was extracted according to Fölch *et al.* (27). Saponification and esterification was carried out according to the method provided by Metcalfe *et al.* (28). Then, 50 mg of extracted oil was saponified with 5 mL of methanolic NaOH (0.5 M) solution by refluxing 10 min at 90 °C. After cooling to room temperature, 2.2 mL 20% BF3–methanolic solution was added, the sample boiled for 3-5 min, again cooled to room temperature and then 1 mL of hexane followed by 1 mL of saturated NaCl solution were added with stirring after each addition. Sodium sulfate was added (1-2 g) and the mixture was shaken to remove the residual water. Finally the upper hexane layer containing the fatty acid methyl esters (FAMEs) was placed in mini-vials. After that, EPA and DHA concentrations were determined by gas chromatography (UNICAM 4600, SB Analytical, UK) equipped with a flame ionization detector (GC-FID) and a BPX-70 fused-silica capillary column (SGE, Melbourne, Australia, 0.22 µm film thickness, 30 m length, 0.25 mm *i.d*.). The operating conditions were as follows: the initial temperature was 50 °C, increased by 5 °C min^-1 ^up to the 160 °C; and with a ramp of 20 °C min^-1^ reached 180 °C and finally 200 °C. The injector and detector temperatures were 250 and 300 °C, respectively. The column head pressure (Helium) was 20 psi. Each FA was identified in its methyl ester form by comparing its retention time with the internal standard (pentadecanoic acid) as described by Sahari *et al*. (29). 

*Phosphorous assay*

The amounts of phospholipids present in the liposomal formulations were determined using colorimetric method (30). Twelve point calibration curves were prepared for DPPC and used for estimation of unknown concentration of lipids in vesicles obtained after each preparation methods. Briefly, 0.1 mL of the liposome suspension (approximately at a concentration of 0.1 mg mL^–1^) was added to 1.9 mL of an aqueous 0.1 M ammonium ferrothiocyanate solution in a test tube. The resulting suspension was mixed with 2 mL of chloroform for 15 s using a vortex, and then centrifuged at 2000× g for 10 min. The upper layer was recovered and analyzed at 488 nm by a UV-vis spectrophotometer (CECIL, UK; model CE 2021).

*Differential scanning calorimetry*

The possibility of any interaction between the omega-3 FAs and liposomes during preparation were assessed by thermal analysis of liposome samples including DPPC bilayer, DHA, and EPA alone as well as combined with each other using DSC (31). A model DSC Shimadzu 60 (Shimadzu Scientific Instruments, Kyoto, Japan) was used to determine melting point and enthalpy for DPPC bilayer with both DHA and EPA or one of them alone. A sample equivalent to approximately 5 mg was placed in aluminum pan and DSC analysis was carried out at a nitrogen flow rate of 20 mL min^-1^ and a heating rate of 5 °C min^-1^ from -5 to 60 °C. An empty aluminum pan was placed on the reference platform. The thermal analysis of sample parameters in the DSC thermogram are the onset temperature (T_0_), the peak temperature or the gel to liquid-crystalline transition (Tm), the endset temperature (Te and T_0_), and enthalpy change of the transition (31, 32). 

*Transmission electron microscopy*

EPA and DHA loaded in liposomes were visualized with negative staining transmission electron microscopy (Ziess, EM10, Germany). The liposome sample was diluted before analysis with phosphate buffer (about 0.05 mg mL^-1^) and then was placed on a 200-mesh formvar copper grid, allowed to adsorb and the surplus was removed by a filter paper. A drop of 1% (w/v) aqueous solution of uranyl acetate was added and left in contact with the sample for 2 min (33). The surplus water was removed and the sample was dried at the room conditions. TEM was performed at operating at an acceleration voltage of 80 KV and viewed under low-dose conditions. 

*Volatile compounds determination*

Volatile compounds were analyzed by headspace liquid phase microextraction procedure (HS-LPME). The internal standard was methanolic solution of 2, 5-dimethylfuran. The extracting solvent was *n*-dodecane, containing 10 mg L^-1 ^internal standard (IS). In a preliminary work, the variables of sample size, extraction temperature, and extraction time were assessed. Sample analysis was performed in different stages. Mixed standard solutions were diluted by double distilled water for preparing the mixing solution amount of each analyte (0.02- 200.00 ng mL^-1^) and after injection to GC/MS their figure of merits were obtained (Table 1).

**Table 1:** Figure of merits for volatile oxidation compounds using the HS-SDME/GC-MS method

**Analyte**	**Regression equation**	**R** ^2^	**RSD (%)**	**Recovery (%)**	**LOD ** **(ng g** ^-1^ **)**	**LOQ ** **(ng g** ^-1^ **)**
Propanal	y=0.002x+0.174	0.998	3.60	98.23	0.008	0.024
Pentanal	y=0.013x+0.2032	0.998	3.33	92.44	0.032	0.074
Hexanal	y=0.043 x+2.122	0.992	4.14	99.53	0.009	0.032
Heptanal	y=0.332 x+2.345	0.998	3.68	94.23	0.030	0.072

Linear range was 0.04-10.00 ng mL^-1^.

Liposome suspension (300 µL) along with 2 g sodium chloride and a micro-stirring bar were placed in a 17.0 mL glass vial sealed with an aluminum crimp cap with a needle pierceable polytetrafluoroethylene/silicon septum. The sample vial was placed into a 40 ºC water bath and stirred at a flow rate of 0.8 mL min^-1^ allowing 10 min for the sample to equilibrate at 40 ºC. The needle of the liquid phase microextraction (LPME) device, containing 3 µL of the extracting solvent composed of *n*-dodecane along with specified concentration of 2,5-dimethylfuran, was inserted into the vial through the septum, and the plunger of the LPME apparatus was pushed down to expose to the vial headspace. After 10 min, the plunger was retracted into the needle assembly, removed from the vial, and transferred to the injection port of the GC unit. GC/MS analysis was carried out using an Agilent technology 7890A gas chromatograph interfaced to an Agilent 5975C inner MSD mass spectrometer (30 m × 0.25 mm; film thickness 0.25 μm, J and W Scientific Agilent Technologies, USA) in EI mode at 70 eV, equipped with a HP-5MS capillary column (34). The mass spectra were obtained in electron-impact mode (EI) at 70 eV and 280 ºC ion source temperature and with a scan range of 30-400 m/z. The oven temperature was set at 40 ºC (3 min as the holding time) and then raised to 280 °C at the rate of 20 ºC min^−1^. Helium was used as the carrier gas at a flow rate of 0.8 mL min^-1^. Three µL of the diluted volatile compounds was injected in the split mode (split ratio was 1:50) and the inlet temperature was held at 280 °C. Identification of volatile compounds in liposome samples were performed using the MSD ChemStation E.0100.237 software for Windows. Peak identification of the volatile compounds was carried out by comparison of the retention times and mass spectra of the eluting compounds to those of the Wiley library (Wiley Nist 05, J. Wiley and Sons Ltd., West Sussex, England).

*Statistical analysis*

The data are presented as mean ± SD of the results from nine independent experiments calculated using Microsoft Excel software (Microsoft, Redmond, WA, USA). Analysis of variance was done for statistical evaluation of the data. The significance of differences between mean values of the preparation methods was determined by the least-significant-difference (LSD) test using the Statistical Analysis System, version 9.1.3 (SAS Institute, Cary, North Carolina, USA). The acceptable probability for a significant difference between means was р < 0.05.

**Table 2:** Size, polydispersity index and zeta potential from various liposomal preparation methods.

**Liposomes**	** Size ** ** (nm)**	**PDI**	**Zeta potential ** **(mV)**
Extruded	99.7 ± 3.5^b^	0.1 ± 0.03^d^	-42.4 ± 1.7^c^
Bath sonicated Probe sonicated	381.2 ± 7.8^a^ 90.1 ± 2.3^c^	0.6 ± 0.03^a^ 0.14 ± 0.02^c^	-43.8 ± 2.4^c^ -36.3 ±1.6^b^
Probe and bath sonicated	87.1 ± 4.1^c^	0.23 ± 0.02^b^	-31.6 ± 1.9^a^

Different superscripts within a column indicate significant differences (p < 0.05).

Results are the average and standard deviations (SD) from nine individual experiments.

## Results and Discussion

**Liposome mean diameter, size distribution and zeta potential determination** Table 2** presents the results obtained from the DLS examinations. The mean size of liposome prepared by probe sonication (90.1 nm) method was smaller than the bath sonication (381.2 nm, p-value <** **0.001) and extrusion (99.7 nm, p-value <** **0.001). Adding the bath sonication method to the probe sonication method did not lead to a significant decrease in the mean size of liposomes (87.1 nm, p-value <** **0.05). Polydispersity is usually expressed as an index of particle diameters in colloidal systems. PDI of liposomes prepared by probe sonication method was lower than those prepared by the bath sonication method (0.14 vs. 0.55, p-value <** **0.001) but higher than those prepared by the extrusion method (0.14 vs. 0.09, p-value=0.004). Adding the bath sonication method to the probe sonication method deteriorated its polydispersity index (PDI= 0.23, p-value <** **0.001).**

Liposomal superfacial characteristics have been reported to be conditioned by the preparation medium. Regardless of the method used, liposomes containing DHA or EPA possess negative charge. The mean absolute value of the zeta potential obtained by all methods was above |30| mV. This indicated that there were acceptable electrostatic repulsive forces between liposomes prepared by different methods under the investigation. Measurement of the zeta potential enables us to predict the storage stability of a colloidal dispersion. The greater the zeta potential, the more likely the suspension is stable; since the charged particles repel each other and as a result overcome the natural tendency to aggregate. It is currently admitted that zeta potentials under 30 mV, optimum >60 mV, are required for full electrostatic stabilization (35). The mean of the zeta potential for liposomes prepared by probe sonication method (-43.8 mV), in terms of absolute value, was higher than those prepared by either the bath sonication (-36.3 mV, p-value <0.001) or extrusion method (-42.4 mV, p-value <0.001). When the bath sonication was combined with probe sonication method, the zeta potential of the liposomes deteriorated (-31.6 mV, p-value >0.01). 

*Assessment of encapsulation efficiency*

**Figure 1.**
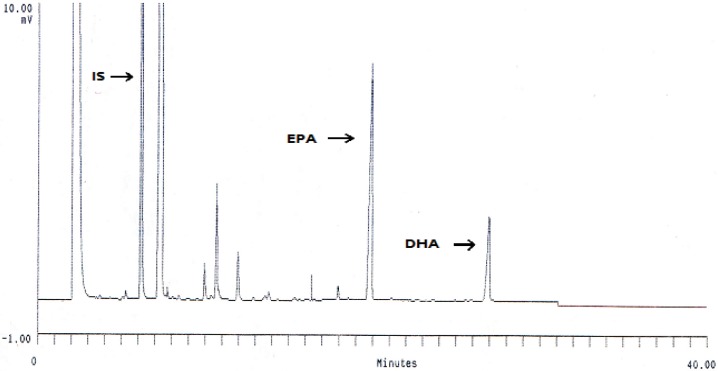
GC separation of DHA and EPA methyl esters in probe sonicated liposome on a BX70 column (Times in min 1= IS RT = 6.25, 2=EPA RT = 19.83, 3 = DHA RT = 26.94).

**Figure 2:**
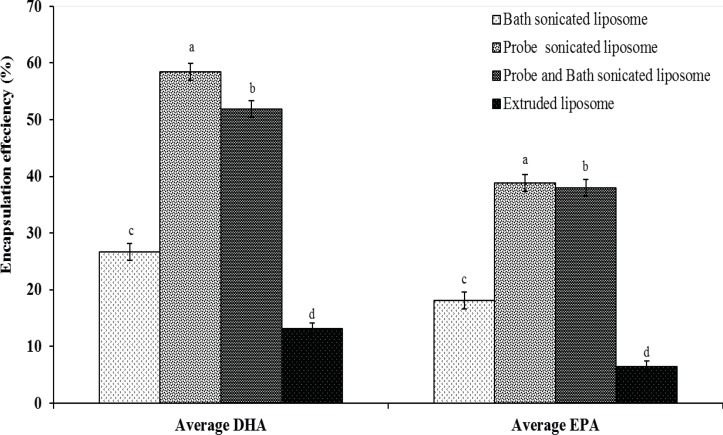
Encapsulation efficiency of DHA and EPA loaded in liposomes from various preparation methods. Different superscripts indicate significant differences (p < 0.05).

Under the chromatographic conditions, GC chromatogram of DHA and EPA encapsulated in liposomes and other fatty acids are indicated in Figure 1. Encapsulation efficiency of DHA and EPA liposomes prepared from probe sonication method was higher than those obtained by other methods (Figure 2). The data indicated that there were no significant differences for EE of DHA and EPA and particle size between probe and combination of probe and bath sonication methods. Regardless of the preparation method, the EE value was higher for DHA than for EPA. Recent studies have shown that DHA have an important role in physical properties of the DPPC bilayer, as it would adopt a tightly pack conformation due to a coiled configuration (36, 37). Optimization loading of lipophilic compounds is not only dependent on the physicochemical properties of these agents, but also on other factors such as the bilayer composition and the method of preparation (15, 38). Accordingly, the loading aspect of contacting the omega-3 FAs with pre-formed liposomes is also an effective method to permit suitable loading of FAs, thereby increasing the EE of DHA and EPA. 

*Phase transition temperature*

**Figure 3:**
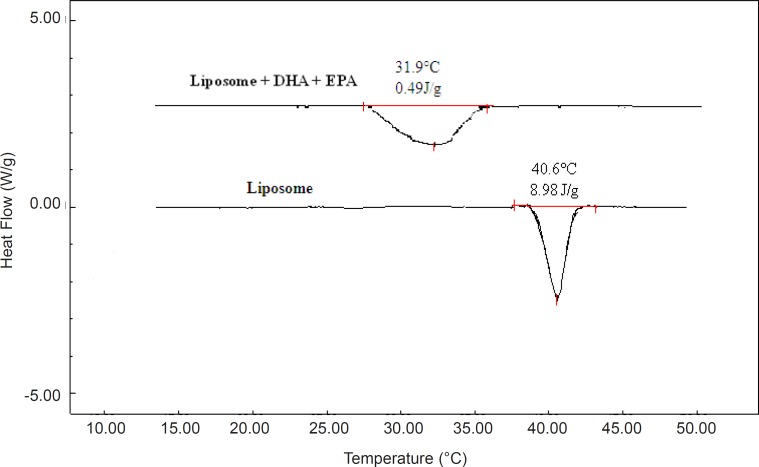
DSC thermograms of DPPC bilayer and DHA and EPA loaded liposomes.

By heating pure phospholipids, the bilayer undergoes a thermotropic gel to liquid crystal phase transition, which is accompanied by an enthalpy change. DSC parameters are shown in Table 3. The phase transition from the gel phase to the liquid-crystalline phase empty MLV made of DPPC (control) was observed at 41 °C. This temperature is close to the phase transition temperature of pure DPPC reported by other researchers. The calorimetric peak of loaded MLV with DHA and EPA showed a shift of phase transition temperature to a lower value and broadened the sharp endothermic peak. The peak temperature (T_m_) of the MLV of DPPC treated with 30 mol% of each or both FAs were 36.7 °C for DHA, 37.4 °C for EPA, and 31.9 °C for mixed fatty acids. Similar results have been described by Onuki *et al.* (31) and Sarpietro *et al.* (39). Encapsulation of DHA and EPA in the liposomes was confirmed by DSC measurements (Figure 3). The endothermic phase transition temperature of the plain liposomes and omega-3 FA-loaded liposomes from probe sonication method was found to be 29.6 and 40.6 ºC, respectively. A negative shift in the transition temperature indicates a strong hydrophobic interaction between omega-3 FAs and the phospholipids forming liposome. The calorimetric measurements on the DHA and EPA liposome interaction and decrease of phase transition temperature and ∆H indicate that this behavior is due to the FA localization inside the lipid bilayer. The presence of omega-3 FAs in MLV or SUV made from DPPC lead to changes in the phospholipid packing by steric restrictions, related to their structure of multiple double bounds, and as a consequence the bilayer stability is reduced. Furthermore, the kinked lipid chains of the unsaturated omega-3 FAs within the bilayer disrupted their normal packing, thus increasing membrane fluidity. The results from thermal analysis including the melting point and enthalpy have an important role for design and optimization of the formulation (36, 39). 

**Table 3:** The effect of DHA and EPA addition on DSC parameters of DPPC bilayer (Mean±SD, n=2).

**DPPC bilayer**	**T**_0_ **(°C)**	**T**_m _ **(°C)**	**T**_e_ **(°C)**	**Enthalpy** ** (Jg** ^-1^ **)**
DPPC	39.3 ± 0.5	40.6 ± 0.4	41.7 ± 0.3	-8.9 ± 0.05
DPPC + DHA	35.7 ± 0.7	36.7 ± 0.7	37.9 ± 0.9	-0.47 ± 0.02
DPPC+ EPA DPPC + DHA + EPA MLV^1^	35.4 ± 0.8 36.6 ± 1.1	37.4 ± 1 35.7 ± 1.3	38.4 ± 1 39.7 ± 1.2	-0.35 ± 0.01 -0.63 ± 0.02
DPPC + DHA + EPA SUV 2	29.7 ± 0.7	31.9 ± 0.9	36.4 ± 1.1	-0.49 ± 0.07

1 Before probe sonication,

2 After probe sonication

*Negative staining TEM images study*

TEM images of nanoliposomes containing DHA and EPA prepared by probe and extrusion methods are close bilayer structures spaced by free internal structure and maintain high structural integrity. The images related to the two other methods show a population of heterogeneous vesicles with increase in size (Figure 4). Briefly, TEM study well-prepared further information about shape and morphology dimensions (40).

**Figure:**
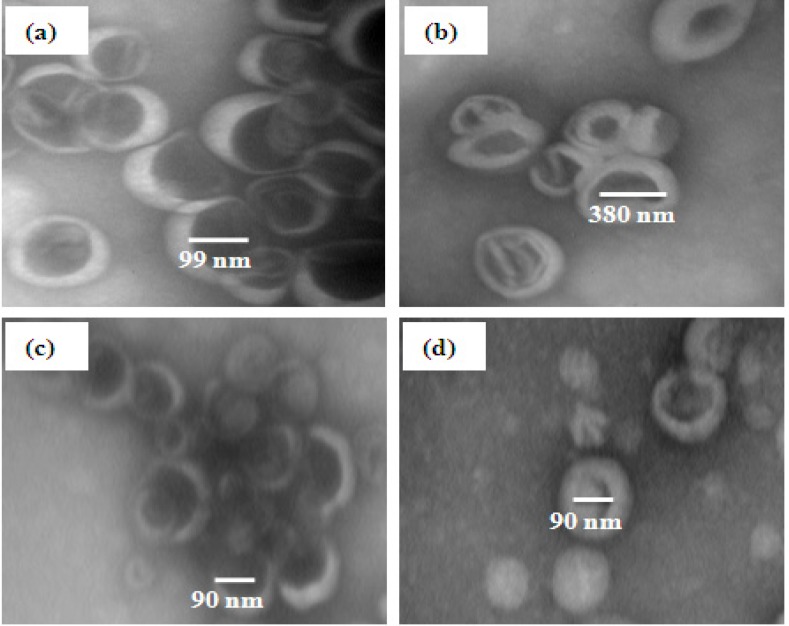
Transmission electron microscopy of DHA and EPA loaded liposomes (a: Extruded; b: Bath sonicated; c: Probe sonicated and d: Bath and probe sonicated liposomes).

*Secondary oxidation products*

**Figure 5:**
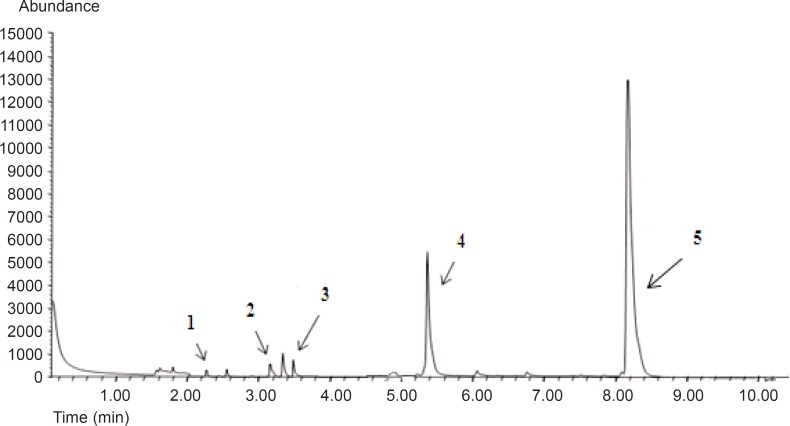
Chromatogram of volatile compounds by GC-MS in probe sonicated liposome. Times in min 1= propanal RT = 2.25, 2=pentanal RT = 3.18, 3 =IS RT = 3.55, 4 = hexanal RT = 5.4, 5 = heptanal RT = 8.25.

DHA and EPA are highly susceptible to oxidation and during processing undergo changes due to their unsaturated nature. Exposure to light, pro-oxidants, and elevated temperature accelerate lipid oxidation and primary oxidative products are formed. Hydroperoxides can decompose to secondary products such as aldehydes, ketones, acids, and alcohol; which causes characteristic off-flavors and odors (41, 42). GC-MS chromatogram of volatile compounds is illustrated in Figure 5. The concentrations of VOCs such as propanal, pentanal, hexanal and octanal; which have been remained in the liposomes were considered as potential indicators of oxidation of omega-3 FAs. As shown in Table 4, the mean concentration of total aldehyde compounds left in preparations after the probe sonication, bath and probe sonication, and extrusion methods were generally higher than those remained after implementing bath sonication methods. The volatile compounds detected are typical oxidation products for omega-3 FAs. Our data revealed that propanal was the main degradation product among VOCs in liposome samples from 96.5 ± 0.11 to 136 ± 0.06 μg/Kg (42). The only exception observed was for hexanal, the mean concentration of which after implementing by all methods was not significant. The oxidizability of DHA and EPA are related to the number of bis- allylic positions (43). The content of EPA and DHA, and their inherent susceptibility to oxidation were most likely the reasons for the levels of VOCs in secondary oxidation products in liposome formulations.

**Table 4:** Volatile compounds from liposomes prepared with different methods expressed in µg/Kg ± SD (n=9).

**Liposomes**	**Propanal **	**Pentanal**	**Hexanal**	**Heptanal **	**Total Volatile Compounds **
Extruded	83.3 ± 4.4^b^	9.3±1.8^a^	10.9± 1.6^a^	4.3±0.7^a^	107.8±6.9^b^
Bath sonicated	59.3 ±2.8^c^	6.5±2.2^b^	10.9±1.2^a^	4.4±0.7^a^	81.2± 4.1^c^
Probe sonicated	81.1±1.1^b^	9.2±1.2^a^	9.7±1.02^b^	3.6± 0.6^b^	103.6 ± 2.2^b^
Probe and bath sonicated	92.8±1.9^a^	10.6±0.8^a^	10.9±0.8^a^	4.5±0.6^a^	118.8±2.3^a^

**Results are the average and standard deviations (SD) from nine individual experiments.** **Different superscripts** **within a column indicate significant differences (** **p** **< 0.05).**

## Conclusion

Liposomes as solubilizing agents can play a key role in addressing the need for improved delivery of polyunsaturated FAs. Optimization loading of DHA and EPA is not only dependent on the physicochemical properties of these compounds, but also on factors such as the bilayer composition and the method of preparation. Probe ultrasound of pre-formed liposomes facilitates a significant loading of DHA and EPA into the nanoliposomal membrane. Probe sonication technique outperformed other three methods. Adding bath sonication to this method did not improve its performance. The DHA/EPA coencapsulated nanoliposomes prepared found to be promising formulation capable of effective reversal, which deserve further investigations.
